# Meanings Attributed to Physical Activity and Changes in Self-Reported and Accelerometer-Measured Physical Activity among Recent Retirees

**DOI:** 10.3390/ijerph192315567

**Published:** 2022-11-23

**Authors:** Miika Tuominen, Sari Stenholm, Pasi Koski, Tuija Leskinen

**Affiliations:** 1 Department of Public Health, University of Turku, 20014 Turku, Finland; 2 Centre for Population Health Research, University of Turku and Turku University Hospital, 20520 Turku, Finland; 3 Department of Teacher Education, University of Turku, 26101 Rauma, Finland

**Keywords:** physical activity, retirement, physical activity promotion, aging

## Abstract

Retirement poses opportunities and challenges for maintaining and adopting physically active habits, which may have major implications for health and functional ability in later life. Qualitative studies suggest that a broad range of meanings of physical activity should be considered when promoting physical activity among retirees. The current study utilized the Physical Activity Relationship (PAR) approach to examine the importance of meanings attributed to physical activity and their associations with physical activity over time. A total of 230 recently retired participants (65.2 years old, 83% women) responded to a 54-item inventory assessing the importance of meanings attributed to physical activity at baseline. Eight meaning dimensions were identified through exploratory factor analysis. Differences in their importance across gender and occupational background were examined using t-tests and ANOVA. Associations between meaning dimensions and self-reported and accelerometer-measured physical activity over 12 months were examined with general linear models. Dimensions defined as Physical Fitness, Positive Mood, and Belonging were positively associated with changes in self-reported and accelerometer-measured physical activity. Additionally, the importance of several meaning dimensions varied across occupational background. In conclusion, physical activity promotion among retirees should focus on physical fitness, positive mood, and social belonging. Furthermore, meanings attributed to physical activity may vary across occupational backgrounds.

## 1. Introduction

Physical activity is important throughout the lifespan to maintain health, well-being and functional ability [1]. However, the levels of physical activity tend to decline with age [2]. Increasing attention has been recently directed towards transitional periods in life as potentially important time windows for promoting physical activity, retirement being the most common transition in late mid-life [3,4].

Life course transitions pose adaptation challenges which may prompt readjustment of established behavioral tendencies [4]. Retirement transition is associated with the loss of work-related roles and social contacts and the need for establishing a post-retirement lifestyle [5,6]. Recreational physical activity engagement is likely to be influenced by the manner in which these challenges are met but may also serve as a method for coping with the adaptation challenges posed by retirement [7,8,9]. Qualitative studies have suggested that the impact of retirement on recreational physical activity tends to be perceived positively [10,11,12,13,14,15,16], with population-based cohort studies observing increases mainly in moderate intensity recreational physical activities, particularly in walking, after retirement [17,18,19,20]. However, the observed increases in recreational physical appear to be relatively minor [21], transient [20,22], and moderated by socioeconomical status [23].

Participants retiring from lower occupational backgrounds have been observed to be less inclined to increase recreational physical activity following retirement [18,23,24,25,26]. Qualitative studies suggest that this may be due to lower valuation of recreational physical activity, lack of perceived need for recreational physical activity due to physically demanding occupation, or viewing retirement as freedom from physical exertion, recreational physical activity included [11,12,13,14,15]. In addition, leisure-time physical activity habits adopted prior to retirement tend to be carried forward [10,11,12,13,14,15,27]. The loss of daily structure due to retirement may represent a barrier to physical activity among those less motivated or accustomed to including recreational physical activity in their everyday life [11,12,13,14]. Yet, the importance of recreational physical activity as a source of daily physical activity is likely to increase after retirement due to the loss of work- and commuting-related physical activity and increased sedentary time [19,24,26,28,29,30]. Consequently, retirement transition also holds potential for increasing the disparities in physical activity with major implications for health with advancing age [31,32]. Thus, identifying the facilitators of recreational physical activity among recent retirees becomes an important task for promoting both the adoption of physically active habits and adaptation to retirement.

Based on two qualitative reviews, the facilitators of recreational physical activity among adults aged 60 or above can be broadly categorized into perceptions of the aging process, health and well-being benefits of physical activity, motivation and habits, social influence, sense of purpose and meaning, and external environment [7,33]. Similar facilitators are commonly identified in qualitative studies focused on recent retirees [10,11,12,13,14,15,16]. In general, the health and well-being related benefits of physical activity tend to be highly regarded by older adults [33]. However, the usefulness of the health-benefit based rationale for physical activity promotion has been recently questioned as it is reliant on providing information on long-term outcomes of which individuals already tend to be relatively well-informed [7,34,35]. More importantly, the functional significance attributed to health and well-being related facilitators is likely to depend on the broader perceptions of the aging itself [11]. Recent retirees with a more negative attitude towards aging may consider the deterioration of health and fitness as inevitable by-products of the aging process and become unmotivated or unsuccessful in their efforts to maintain or uptake physical activity for the sake of health and well-being following retirement [10,11,12,15]. On the other hand, physical activity may be considered as an important way to maintain health and well-being in retirement even in the presence of physical health-related barriers among retirees more positively oriented towards aging [11,12,15]. Thus, it has been suggested that further attention should be focused on the broad range of meanings attributed to physical activity, particularly during transitional phases [7].

Previous qualitative studies focused on retirement and physical activity have suggested that engagement in recreational physical activity may contribute to a sense of purpose by providing structure and meaningful content in the abruptly altered daily schedules of recent retirees [11,12,14,15]. Recreational physical activity can also provide a context in which to meet challenges, experience competence and learn new skills, which may foster an overall sense of independence and confidence that can become challenged by retirement [7,15,33], particularly among men [12]. Furthermore, recreational physical activity may serve as a context for social engagement, which may help to replace work-related social ties lost due to retirement [15]. While often identified as more salient for women, social factors are suggested to be supportive of recreational physical activity engagement to the extent that they enhance belonging, provide encouragement and facilitate social connectedness [10,12,13,14,15]. In addition, some common barriers to recreational physical activity, such as lack of time and energy, are likely to reduce in importance following retirement [14], while facilitators inherent in the environment, such as affordability, proximity and access to facilities are likely to remain influential [7,13,33].

While qualitative studies provide important insights about the facilitators of recreational physical activity following retirement, they cannot be used to evaluate the strength or generalizability of the associations suggested. Furthermore, it is difficult to distinguish between factors associated with the levels of recreational physical activity and the facilitators of changes in recreational physical activity based on qualitative evidence. Thus, some facilitators of physical activity among recent retirees identified by previous qualitative studies may represent reasons for why people are physically (in) active in general, rather than facilitators and barriers of change as such. For example, a previous longitudinal quantitative study focused on recent retirees failed to confirm several associations between changes in leisure-time physical activity and individual-, social- and environmental-level facilitators suggested by qualitative findings [36]. Of the facilitators examined, only self-efficacy was found to predict changes in leisure-time physical activity among recent retirees, whereas no associations with perceived benefits, perceptions of retirement, social support, or other social or environmental facilitators were observed [36]. However, quantitative studies focused on the facilitators of physical activity following retirement transition continue to be scarce. There is a need for further quantitative prospective studies in order to identify facilitators to target when promoting physical activity among recent retirees.

In the current study, an approach based on the Physical Activity Relationship (PAR) approach was adopted [37,38,39]. PAR has a sociological foundation and views recreational physical activity as a facet of the socially constructed reality. Meaning is a central concept in PAR that is further elaborated elsewhere [37,38]. Briefly, it refers to both the extent to which an individual recognizes and attributes personal significance to meanings associated with recreational physical activity within a given sociocultural context. These personal attributions, in turn, are assumed to be dynamic and produced through encounters with contexts of recreational physical activity throughout the lifespan via the processes of socialization and internalization. Thus, the personal importance attributed to various meanings of recreational physical activity is assumed to reflect both the familiarity with and personal valuation of recreational physical activity.

A central hypothesis of PAR is that the personal importance of meanings attributed to recreational physical activity is predictive of the levels of physical activity. In PAR, the meanings of recreational physical activity are categorized into broader dimensions, generally characterized as competition and accomplishment, health and healthiness, social aspects, expressive aspects, play and joy, and aspects related to the self [38]. There is a degree of theoretical and empirical divergence regarding the number and nature of the underlying meaning dimensions (e.g., [38,40,41,42,43]). However, the central hypothesis of the positive association with the strength of attributed meanings and levels of physical activity has found empirical support among children and adolescence [38,44], young adults [42], and across the lifespan [39]. The physical activity meaning dimensions suggested by PAR also align with the categories of facilitators of physical activity identified in qualitative reviews [7,33]. Thus, the meaning-based approach of PAR is well suited for quantitatively examining associations suggested by previous qualitative research on the facilitators of physical activity following retirement [10,11,12,13,14,15,16].

The purpose of the current study was to examine whether meanings attributed to physical activity were associated with changes in both self-reported and accelerometer-measured physical activity among recent retirees. First, we aimed to identify the physical activity meaning dimensions among recent retirees as previous studies using the PAR inventory have been mainly focused on younger populations [38,39,42,44]. Second, we examined the overall importance of the physical activity meaning dimensions across genders and occupational backgrounds as differences were expected based on previous qualitative studies [11,12,13,14,15]. Third, we examined the extent to which the importance attributed to different physical activity meaning dimensions at the baseline were associated with changes in physical activity over 12-month follow-up using both self-reported and accelerometer-measured physical activity outcomes. In addition, previous PAR based studies have been conducted mainly among younger study populations with cross-sectional data based on self-reported physical activity [38,39,42,44] which is subject to number of limitations [45]. Thus, the current study also serves as empirical test for the central hypothesis of the PAR approach while using a prospective study design with self-reported and accelerometer-measured physical activity outcomes among recent retirees.

## 2. Materials and Methods

### 2.1. Participants

The current study utilizes data from a 12-month follow-up collected in the context of a technology-based physical activity intervention among a community-based sample of recently retired participants (Enhancing Physical Activity and Healthy Aging among Recent Retirees, REACT randomized controlled trial) [46]. The REACT trial examined the impact of a consumer-based activity tracker on the levels of daily activity among participants who had recently transitioned to full-time retirement. The sampling of the REACT trial was targeted at Finnish public sector workers whose estimated statutory retirement date was between January 2016 and April 2019 and who were living in Southwest Finland in 2017. The inclusion criteria included the actual date of retirement between January 2016 and December 2018, ability to walk for 500 m uninterrupted, no current post-operative state or known surgeries within the next six months, no malign cancer or recent myocardial infraction, and basic knowledge and ability to use computer and Internet at home [46].

Of 1475 eligible individuals (79% women) contacted, 272 expressed their willingness to participate in the trial. The 272 respondents were more likely to be women (82% vs. 78%) and highly educated (37% vs. 20%) when compared with the 1203 non-respondents. Of the 272 individuals expressing interest, 231 fulfilled the inclusion criteria and were able to participate in the trial (average time since retirement at baseline 1.2 years, SD 0.5) [46]. For the present study, one participant of the 231 was excluded due to missing questionnaire data at the baseline.

The REACT trial was approved by the ethics Committee of Hospital District of Southwest Finland (107/1801/2017) and registered at ClinicalTrials.gov: NCT03320746. Informed consent for participation was obtained from all participants.

### 2.2. Procedures

The data were collected using electronic questionnaires and wrist-worn accelerometers (Actigraph wGT3X-BT, ActiGraph LLC, Pensacola, FL, USA) at baseline and at 12-month follow-up. In the REACT trial, 231 participants were randomized to intervention and control groups and allocated in five waves of data collection distributed throughout the year. The baseline data were collected prior to randomization. The intervention and control groups were not distinguished for the purposes of the study at hand as no differences in physical activity outcomes were observed between the intervention and control groups during the 12-month follow-up [46].

### 2.3. Measures

The meanings attributed to physical activity were assessed with a 54-item inventory based on the PAR approach [37,38]. The 54-item inventory has been previously used among university-level students in Finland [42], while similar questionnaires with varying number of items have been used among children and adolescents [38,44]. The questionnaire presents participants with 54 items related to recreational physical activity, such as “feeling good”, “new experiences”, “cooperation and support”. The participants are asked (“How important the following things are for your recreational physical activity engagement?”) to rate the personal importance of each item on a five-point scale (1 = completely insignificant, 2 = fairly insignificant, 3 = neutral, 4 = fairly important, 5 = very important).

The levels of self-reported leisure-time physical activity (LTPA) were assessed at the baseline and at 12-month follow-up with a four-item questionnaire assessing the amount of average weekly hours spent in recreational physical activities corresponding with (a) walking, (b) brisk walking, (c) jogging and (d) running during the past three months [47]. The response options were “Not at all”, “Less than 30 min”; “Approximately one hour”, “Two to Three hours” and “Four hours or more”. The responses were coded as hours as follows: 0, 0.5, 1, 2.5, and 4, respectively, and aggregated to represent the weekly hours of self-reported recreational physical activity.

The average daily minutes spent in total (Total PA) and moderate-to-vigorous physical activity (MVPA) were measured with wrist-worn accelerometers (Actigraph wGT3X-BT, ActiGraph LLC, Pensacola, FL, USA) at baseline and 12-month follow-up. The accelerometers were delivered simultaneously with the study questionnaires at both time points. The participants were requested to wear the accelerometer on their non-dominant wrist for eight days and seven nights on both measurement points. A measurement day was considered valid if the accelerometer was worn for a minimum of ten hours per day. At least four valid days were required for calculating the average daily minutes within a given week. A more detailed description of the accelerometer data processing is described elsewhere [46].

Information on age, gender (man/woman) and occupational background (high/middle/low) was derived from the Pension Institute’s register. Occupational background was assessed with International Standard Classification of Occupations (ISCO) and categorized into three levels as follows: “High” including managers and professionals, such as teachers and doctors (ISCO classes 1–2), “Middle” including associate professionals, such as registered nurses and clerical workers (ISCO classes 3–4), and “Low” including manual and service workers, such as practical nurses, cooks and maintenance workers (ISCO classes 5–9).

Self-rated health was assessed on a five-point scale included in the baseline questionnaire (1 = good, 2 = rather good, 3 = average, 4 = rather poor, 5 = poor). Body mass index (BMI, kg/m^2^) was calculated from the measured body height and weight at the baseline.

### 2.4. Statistical Analysis

#### 2.4.1. Exploratory Factor Analysis

An exploratory factor analysis was conducted to identify the physical activity meaning dimensions and items to retain for further analyses. A data-driven approach was adopted as previous studies using the PAR approach have been mainly conducted among younger populations [38,42,44]. Principal axis factoring with oblique rotation (promax) was used due to skewed distribution in some of the items and as the extracted factors were expected to be correlated. Pairwise exclusion was used to deal with missing values as the total amount of missing values was low (0.93% of possible values missing). The exploratory factor analysis was conducted using SPSS (Version 28.0; IBM Corp., Armonk, NY, USA) whereas further analyses were conducted with SAS (version 9.4; SAS Institute Inc, Cary, NC, USA).

#### 2.4.2. Physical Activity Meaning Dimensions and Background Characteristics

The average scores representing the importance of each physical activity meaning dimension identified in the exploratory factor analysis were summarized descriptively with means and standard deviations. Between-gender differences in the importance of the meaning dimensions were examined with independent samples t-tests. The differences in the importance of the meaning dimensions across occupational background were examined with ANOVA. The effect sizes were quantified with Cohen’s d with pooled standard deviations for t-tests and omeqa squared (ω^2^) for ANOVA. Two-tailed *p*-values below 0.05 were considered significant for all analyses.

#### 2.4.3. Associations between Physical Activity Meaning Dimensions and Physical Activity

Pearson correlation was used to examine the unadjusted associations between physical activity meaning dimensions and physical activity outcomes at baseline. General linear models were used to examine the association of physical activity meaning dimensions with the levels of physical activity at baseline and changes in physical activity over 12 months while adjusting for covariates. Separate models were fitted for self-reported LTPA and accelerometer-measured total PA and MVPA. Standardized values (M = 0, SD = 1) were used in all analyses to foster the comparison of the associations across different physical activity meaning dimensions and physical activity outcomes. Square root transformed values of total weekly hours of self-reported LTPA and accelerometer-measured average daily minutes of MVPA were used due to positively skewed distributions. Variables representing the importance of each physical activity meaning dimension were entered separately in the models as a predictor as the dimensions were assumed to be correlated. The associations of physical activity meaning dimensions and changes in physical activity were examined by including the physical activity outcome at 12 months as an outcome variable while adjusting for baseline. All models included gender, occupational background and self-rated health as categorical and age and body mass index as continuous covariates. In addition, intervention group was added as a categorical covariate when changes in physical activity over time were examined. The models with accelerometer-measured outcomes were further adjusted with wear time of the device. The normality of the residuals was examined visually.

## 3. Results

### 3.1. Participants

At the baseline, the participants were 65.2 (SD 1.1) years old, mainly women (83%), relatively healthy (83% with good or rather good self-rated health) and reported an average of 5.7 (SD 2.9) hours of weekly leisure-time physical activity. On average, the participants accumulated 53 average daily minutes (SD 30) of accelerometer-measured MVPA and 4 h and 35 min (SD 1 h 31 min) of daily Total PA. Of 230 initial participants, five dropped out of the study during the 12-month follow-up. The participant characteristics are presented below in Table 1.

### 3.2. Physical Activity Meaning Dimensions

The responses to the individual 54 items in the meaning inventory are presented in a Supplementary Table S1 with means, standard deviations and the percentage of participants rating a given item fairly or very important (Supplementary Table S1). The highest ratings were given to “maintaining health”, “restoration”, “nature”, “feeling good”, “joy”, and “improving fitness”, which were all rated as important or very important by ≥90% of the respondents.

Prior to conducting the exploratory factor analysis, three items from the 54-item inventory were removed due to high bivariate correlation (“winning/success” removed due to high correlation with “competition”, r = 0.84), low correlation with other items (all bivariate correlations for “making social contacts” r ≤ 0.23), or overlap in content (“regular schedule” removed due to conceptual overlap with “regularity”). Thus, the exploratory factor analysis was conducted with 51 items. Items having extracted communalities below 0.40 were excluded and the analysis was re-run with the 45 remaining items. Eight factors explaining 61.0% of the initial variance in the 45 items were identified based on theoretical consideration, scree-plot and a minimum requirement of at least two loadings of ≥0.50 per factor. For the final solution, eight factors with 32 items having a minimum loading of 0.50 were retained. Cronbach alphas for the eight identified dimensions were acceptable and ranged from 0.65 to 0.86. Finally, the observed scores of the retained items within a given meaning dimension were averaged for further analyses. In case of missing values in the items, an average score based on the existing responses was calculated as each participant with missing values had responded to on minimum half of the items within any given dimension. The identified meaning dimensions were named as Trends and Status, Mental Well-Being, Physical Fitness, Positive Mood, Belonging, New Experiences, Achievement, and Practical Facilitators. The final solution of the exploratory factor analysis is presented in Table 2.

### 3.3. Background Characteristics and Physical Activity Meaning Dimensions

Participants’ ratings of the importance of physical activity meaning dimensions are summarized across gender and occupational background in Table 3. On average, the participants rated the highest importance for Positive Mood (Mean 4.35, SD 0.58) and Physical Fitness (Mean 4.28, SD 0.59), followed by Practical Facilitators (Mean 3.70, SD 0.85), Mental Well-Being (Mean 3.41, SD 0.73), New Experiences (Mean 3.41, SD 0.78) and Belonging (Mean 3.22, SD 0.88). Achievement was rated as relatively low in importance (Mean 2.80, SD 0.78) whereas Trends and Status was found fairly insignificant (Mean 1.74, SD 0.76).

Women rated higher importance for Practical Facilitators (Mean difference 0.58, 95% CI 0.29 to 0.87), Belonging (Mean difference 0.44, 95% CI 0.14 to 0.74), Mental Well-Being (Mean difference 0.34, 95% CI 0.10 to 0.59), and Positive Mood (Mean difference 0.24, 95% CI 0.04 to 0.44) when compared with men. No statistically significant between-gender differences were observed in the importance of Trends and Status, Physical Fitness, New Experiences, or Achievement.

Across occupational backgrounds, statistically significant differences were observed across all physical activity meaning dimensions, apart from Positive Mood and Physical Fitness. Participants with low occupational background rated higher importance for Mental Well-Being when compared with participants with both middle (Mean difference: 0.34, 95% CI 0.05 to 0.62) and high occupational background (Mean difference: 0.52, 95% CI 0.26 to 0.78). Similarly, Achievement was found more important by participants with low occupational background when compared with participants with both middle (Mean difference: 0.34, 95% CI 0.05 to 0.62) and high occupational background (Mean difference: 0.60, 95% CI 0.32 to 0.87). In addition, participants with low occupational background rated higher importance for Practical Facilitators (Mean difference: 0.39, 95% CI 0.07 to 0.70), Belonging (Mean difference: 0.37, 95% CI 0.05 to 0.70), and New Experiences (Mean difference: 0.30, 95% CI 0.01 to 0.59) when compared with participants with high occupational background. Due to violation of the homogeneity of variance assumption as indicated by a significant Levene’s test, differences in the importance of Trends and Status across occupational categories were examined using Kruskal–Wallis test (H (2) = 15.6, *p* = <.001) with the Dwass, Steel, Critchlow–Fligner method for multiple comparisons. Although Trends and Status was rated unimportant in general, participants with low occupational background tended to rate higher values when compared with participants with both middle (*p* < 0.001) and high (*p* < 0.001) occupational background.

The distribution of occupational backgrounds between genders was examined to assess potential confounding in the observed differences. Men (53.4% High, 23.1% Middle, 23.1% Low) were observed to be more likely to have a high occupational background when compared with women (35.1% High, 27.8% Middle, 37.2% Low), albeit the difference in distributions was not statistically significant (χ^2^ (2, *n* = 230) = 5.09, *p* = 0.078). Nevertheless, further sensitivity analyses were conducted to compare the importance of the meaning dimensions across occupational backgrounds among women. Due to the low number of men in the sample, the sensitivity analyses were limited to women. Among women, the results remained similar with statistically significant differences across occupational backgrounds observed in the importance of Mental Well-Being (F_2,188_ = 9.78, *p* < 0.001), Achievement (F_2,188_ = 15.95, *p* < 0.001), Practical Facilitators (F_2,188_ = 4.09, *p* = 0.018), Trends and Status (H (2) = 37,7, *p* < 0.001) and New Experiences (F_2,188_ = 3.05, *p* = 0.050). However, no statistically significant differences in the importance of Belonging across occupational backgrounds were observed among women (H (2) = 4.0, *p* = 0.13). Thus, the differences observed across occupational backgrounds in Belonging are likely to be partly due to the unbalanced gender distributions across occupational backgrounds. In general, the observed differences remained similar among women with women with low occupational background rating the highest importance for the physical activity meaning dimensions mentioned above.

### 3.4. Associations of Physical Activity Meaning Dimensions with Self-Reported LTPA

The correlations at baseline and the results of the covariate adjusted general linear models are presented in Table 4 for self-reported LTPA. Correlations between physical activity meaning dimensions and the levels of self-reported LTPA at baseline were positive and mainly small or small-to-moderate (*r* ranging from 0.07 to 0.19). After adjusting for covariates, New Experiences (B = 0.21, *p* = 0.002), Physical Fitness (B = 0.18, *p* = 0.005), Belonging (B = 0.17, *p* = 0.015), Mental Well-Being (B = 0.14, *p* = 0.033), and Positive Mood (B = 0.13, *p* = 0.047) were found to be positively associated with the levels of self-reported LTPA at baseline. However, only Physical Fitness (B = 0.18, *p* = 0.004), Positive Mood (B = 0.18, *p* = 0.004), and Belonging (B = 0.14, *p* = 0.030) were positively associated with changes in self-reported LTPA over 12 months after adjusting for covariates.

### 3.5. Associations of Physical Activity Meaning Dimensions with Accelerometer-Measured Total PA

The correlations at the baseline and the results of the covariate adjusted general linear models are presented in Table 5 for accelerometer-measured total PA. Correlations between physical activity meaning dimensions and the levels of accelerometer-measured total PA at the baseline were positive and mainly weak or small (*r* ranging from 0.04 to 0.15). No statistically significant associations with physical activity meaning dimensions and the levels of accelerometer-measured total PA at the baseline were observed after adjusting for covariates. However, Practical Facilitators (B = 0.15, *p* = 0.002), Belonging (B = 0.12, *p* = 0.012), and Physical Fitness (B = 0.11, *p* = 0.021) were positively associated with changes in accelerometer-measured total PA over 12 months after adjusting for covariates. Furthermore, Positive Mood (B = 0.09, *p* = 0.062) was also observed to be positively associated with changes in accelerometer-measured total PA, albeit the association was small and not statistically significant.

### 3.6. Associations of Physical Activity Meaning Dimensions with Accelerometer-Measured MVPA

Correlations between physical activity meaning dimensions and the levels of accelerometer-measured MVPA at the baseline were varied and mainly weak (*r* ranging from −0.06 to 0.10). No statistically significant associations were observed with the physical activity meaning dimensions and the levels or changes in accelerometer-measured MVPA after adjusting for covariates. However, a small positive association was observed with Physical Fitness and changes in MVPA over 12 months (B = 0.09, 95% CI 0.00 to 0.19, *p* = 0.061) although the association was small and statistically non-significant (Supplementary Table S2).

## 4. Discussion

The current study utilized Physical Activity Relationship (PAR) approach [37,38] to quantitatively examine associations with the facilitators of recreational physical activity and changes in physical activity suggested by previous qualitative studies focused on recent retirees. The main purpose of the study was to examine the importance of meanings attributed to physical activity and their association with changes in physical activity among recent retirees. Furthermore, associations with both levels and changes in physical activity were examined in order to compare the cross-sectional and longitudinal associations using both self-reported and accelerometer-measured metrics of physical activity.

Eight physical activity meaning dimensions were identified through exploratory factor analysis: Positive Mood, Physical Fitness, Practical Facilitators, Mental Well-Being, New Experiences, Belonging, Achievement and Trends and Status. The identified dimensions were in agreement with those identified in previous studies based on the PAR approach conducted among children and adolescence [38,44], young adults [42], and across the lifespan [43], notwithstanding some differences in the number and characterization of the meaning dimensions.

Physical Fitness and Positive Mood were observed to be highly important across gender and occupational background and positively associated with changes in both self-reported LTPA and accelerometer-measured Total PA. These observations are in agreement with previous studies suggesting that older adults in general tend to be well aware of, and highly value, the health and well-being related benefits of physical activity [7,33]. However, the relevance of health and well-being related benefits for promoting physical activity has been questioned [7,34,35]. In addition, a previous longitudinal study focusing on recent retirees observed no associations between perceived health and well-being benefits of physical activity and changes in physical activity [36]. Yet, we found Physical Fitness and Positive Mood to be both highly important and positively and consistently associated with changes in physical activity over 12 months. This could be partly attributable to the items of Physical Fitness, i.e., “improving fitness”, “maintaining health”, and “physical exertion”, placing emphasis on the maintenance of health and functionally oriented fitness [34]. In addition, Positive Mood was defined by items such as “joy” and “restoration”, which reflect the immediate emotional outcomes associated with physical activity engagement that are likely to differ from the more cognitively oriented perceived well-being benefits. However, the high relevance of Physical Fitness and Positive Mood could also reflect the relatively healthy and active sample of our study for whom the associated meanings may be particularly salient [11,12,15].

Positive Mood was rated highly important in general, but the importance of *Mental Well-Being* was markedly lower and varied across gender and occupational background. In comparison to Positive Mood, Mental Well-Being could be defined by coping with negative emotions, characterized by items such as “unwinding” and “alleviating stress”. Both enjoyment and psychological well-being related motives for physical activity have been previously identified as highly salient among adults aged 60 or above [7,33], with higher importance attributed to psychological well-being by women [48]. However, in addition to gender, we observed the importance of Mental Well-Being to vary strongly across occupational backgrounds. It has been previously observed that those with higher socioeconomic status are more likely to benefit from retirement in terms of improved well-being [31]. The higher importance attributed to Mental Well-Being by participants with a low occupational background may thus indicate higher relevance of recreational physical activity as a method for coping with adaptation challenges posed by retirement [8,9]. For example, in a previous study focused on recent retirees, positive perceptions of retirement were observed to be positively associated with changes in leisure-time physical activity only among retirees with low educational background [36]. Nevertheless, Mental Well-Being was not found to be associated with changes in physical activity over 12 months, suggesting that when the well-being related benefits of physical activity are promoted among recent retirees, they should be primarily framed in terms of the immediate positive emotional outcomes of physical activity, such as those characterized by Positive Mood.

Practical Facilitators was reliant only on two items, namely “proximity” and “affordability”, which are commonly identified environmental facilitators or barriers to physical activity among adults aged 65 or above [49]. The relatively high overall importance of Practical Facilitators may thus be attributable to its relevance for walking and indoor physical activities, which count among the preferred forms of recreational physical activity among retirement aged individuals [50]. The higher importance attributed to Practical Facilitators by participants with a low occupational background may reflect socioeconomical differences in resources and possibilities offered for recreational physical activity by the neighborhood environment [49,51,52]. However, Practical Facilitators was observed to be positively associated only with changes in accelerometer-measured Total PA over the 12 months, which may reflect the higher relevance of environmental facilitators for non-exercise related physical activities [53,54]. Thus, the higher importance attributed to Practical Facilitators by recent retirees with a low occupational background may also indicate a higher preference for non-exercise related physical activities among retirees with lower socioeconomical statuses [15,51,55]. However, the observed association with Practical Facilitators and changes in total PA could also be due to the high proportion of women in the sample, for whom the environmental facilitators may have higher relevance [56,57].

Social facilitators are often cited as salient particularly for women in qualitative studies focused on recent retirees [10,12,15]. However, the importance of Belonging, while higher than among men, was observed to be relatively low also among women. The previous qualitative studies may have overestimated the importance of social facilitators as many retirement-aged adults may hold a preference for solitary exercise [50]. Alternatively, the importance of social facilitators may be better captured by constructs other than Belonging, defined by items such as “being in a group” and “like-minded others”. For example, “making social contacts” and “spending time with significant others” were rated highly important as individual items but they were not loading with the Belonging dimension in the exploratory factor analysis (Supplementary Table S1). Consequently, some relevant dimensions of social facilitators are likely to have remained uncaptured by the inventory used in the current study [58]. Yet, we observed Belonging to be positively and consistently associated with changes in both self-reported LTPA and accelerometer-measured Total PA over 12 months. This is in contrast to a previous study observing no associations between changes in leisure-time physical activity and a range of social facilitators (including social modeling, social support, and social cohesion of the neighborhood) among recent retirees [36]. The discrepancy with our findings is likely to be attributable to the manner in which the social facilitators were operationalized as the items included under Belonging reflect a degree of togetherness missing from the social facilitators examined in the previous study. However, our findings could also be partly attributable to the high proportion of women in our sample, for whom the association with social facilitators and physical activity may be particularly salient [10,12,15].

No gender differences were observed in the importance of skills, challenge and achievement related dimensions of New Experiences and Achievement although they have been suggested to be particularly salient for men [12]. Rather, New Experiences, characterized by items such as “learning new skills” and “versatility”, was observed to be moderately important, whereas Achievement, characterized by items such as “sense of competence” and “aiming for better performances” was found to be relatively unimportant in general. Furthermore, New Experiences was observed to be positively associated with the levels of self-reported LTPA at the baseline. Thus, the potential contribution of recreational physical activity to fostering a sense of purpose and meaning through challenge and achievement among recent retirees is likely to be better captured by the possibilities it offers for exploring new experiences and learning new skills [12,15]. However, New Experiences was not observed to be associated with changes in physical activity over 12 months, suggesting that it may serve to facilitate physical activity among recent retirees already physically active. Finally, Trends and Status was found neither important nor associated with physical activity on any metric suggesting that the relevance of various exercise-related trends among recent retirees is minimal.

Retirement transition has been recently identified as an important time point for promoting physical activity among older populations [3,4]. However, the current knowledge regarding the facilitators of increased physical activity following retirement transition is limited and mainly reliant on qualitative evidence [10,11,12,13,14,15,16]. Furthermore, the extent to which the qualitative findings can be replicated by quantitative designs is currently unclear [36]. A key challenge in examining the associations suggested by qualitative studies using quantitative designs is the manner in which the central concepts are operationalized. In contrast to a similar earlier study [36], we were able to identify several associations suggested by previous qualitative findings using both self-reported and accelerometer-measured metrics of physical activity. This is likely to be attributable to our reliance on the meaning-based PAR approach [37,38] although our sample of mainly of healthy and physically active women is also likely to have influenced our observations. However, the PAR inventory is well-aligned with a broad range of facilitators identified by previous qualitative studies [7,33]. Thus, the PAR may provide a well-suited tool for quantitatively examining the associations suggested by qualitative studies focused on retirement and physical activity. We were able to confirm and extend several findings associated with the facilitators of physical activity following retirement suggested by previous qualitative studies [10,11,12,13,14,15,16]. More specifically, our findings suggest that among recent retirees, the health-related benefits of physical activity should be promoted with a focus on maintaining health and fitness. The well-being related benefits should be primarily framed in terms of the immediate emotional outcomes of physical activity engagement. Social facilitators, in turn, are likely to be particularly salient when operationalized as social belonging experienced in the context of recreational physical activity. Furthermore, social belonging may be particularly relevant to those retirees aiming to increase their levels of physical activity. Our findings also suggest that practical facilitators or barriers inherent in the environment may be more relevant to overall than recreational physical activity as associations were observed only with changes in total PA. In addition, while experiencing challenge, achievement and learning new skills have been identified as facilitators of physical activity particularly among men [12,15], our findings suggest that these kinds of facilitators may be more relevant to those already physically active and equally important across genders.

Furthermore, we observed differences across categories of occupational background in the importance of several physical activity meaning dimensions, including Mental Well-Being, Practical Facilitators, New Experiences, Achievement, and Trends and Status. Low socioeconomical status has been previously found to be associated with lower inclination towards recreational physical activity following retirement [18,24,25,26], with qualitative studies attributing this observation to lower valuation and motivation towards recreational physical activity [11,12,13,14,15]. However, we observed an opposing trend. Moreover, differences were observed in the higher importance attributed to a broad range of physical activity meaning dimensions by participants with low occupational background. This observation could be due to different preferences for recreational physical activity among those retiring from physically demanding occupations when compared with those retiring from sedentary occupations [11,13,15]. In addition, recent retirees with lower socioeconomical status may be more likely to place higher value on purposeful physical activities [15,55], which could also be reflected in the meanings attributed to physical activity. Thus, further research on the varying importance of different preferences and facilitators of physical activity across socioeconomical backgrounds among recent retirees is warranted [59].

Overall, the current study highlights the importance of quantitatively examining the associations suggested by qualitative findings on retirement and physical activity. Although this can be achieved through different operationalizations of the central concepts [36], the meaning-based PAR approach appears well-suited. Furthermore, we found support for the central hypothesis of the PAR approach using both self-reported and accelerometer-measured physical activity outcomes. Thus, PAR appears to offer an interesting approach for examining the facilitators of physical activity that should be further developed.

### Strengths and Limitations

This study is the first to quantify the relative importance of a broad range of meanings attributed to physical activity and changes in physical activity among recent retirees. However, there are a number of important limitations.

First, the sample of the current study was small for the purposes of exploratory factor analysis although relatively strict criteria were used to identify the number of factors and items to retain. Furthermore, due to the low number of men, we were not able to examine the factor structure separately for men and women. Thus, the identified factor structure may be biased towards women. In the previous PAR-based studies focused on younger populations, differences in the importance of meanings of physical activity between genders have been commonly observed [38,42,43]. However, the extent to which similar factor structures are applicable across genders is unclear as this has not been formally examined in the previous PAR-based studies [42,43]. Nevertheless, the purpose of the exploratory analysis in the current study was not to establish the factor structure of the PAR inventory among recent retirees in general but to provide a data-driven justification for categorization of the individual items for further analyses. The eight-factor solution with 32 of the initial 54 items retained is comparable to the 37 items retained in a previous study using the 54-item inventory [42], and with 34 items included in the most recent version of the inventory [38]. Nevertheless, there were some differences observed in the number of identified physical activity meaning dimensions and the loadings of individual items when compared with previous studies using the PAR approach [38,42,43,44]. This is likely to depend partly on the variation in the questionnaires used, sample sizes and analytical choices across previous studies. However, the observed differences may also be due to variations in the relevance and interpretation of the individual items across different aged populations, which is consistent with the assumptions of PAR [43]. Yet, some individual items receiving high ratings were not included in the final factor solution due to low communalities or loadings or both (Supplementary Table S1). Thus, further refinement of the inventory is likely to be required.

Second, the analytical power of our study was limited. Previous studies based on PAR with larger samples have observed a general association with the number of meanings found important and levels of physical activity, regardless of the physical activity meaning dimension examined [38,39,42,44]. Thus, it is possible that given a larger sample with increased power, we would have observed all the dimensions to be associated with physical activity outcomes. However, the previous studies based on PAR have been cross-sectional and reliant on self-reported measures of physical activity. The prospective design of our study allowed us to examine the association of physical activity meaning dimensions with levels and changes using both self-reported and accelerometer-measured physical activity over 12 months. Notwithstanding the high levels of uncertainty evidenced by the wide confidence intervals, differences in the strength of the associations were observed across physical activity meaning dimensions, suggesting that some dimensions may have higher relevance for recreational physical activity engagement than others, particularly when changes in physical activity are considered. Furthermore, the observed associations tended to be stronger for self-reported LTPA, which is likely to be attributable to common method bias. However, the strongest associations remained similar for both changes in self-reported LTPA and accelerometer-measured total PA. Yet, accelerometer measured MVPA was not observed to be associated with any of the physical activity meaning dimensions, apart from a small and statistically non-significant association with Physical Fitness. As the meaning inventory aims to assess meanings relevant particularly for recreational physical activity, the absence of observed associations with MVPA reduces the trustworthiness of the findings. There are some known limitations related to using wrist-worn accelerometers, as they fail to recognize activities where the wrist remains relatively immobile with lower body movement, such as cycling. However, the observed associations may also indicate a preference for lower intensity recreational physical activities among recent retirees, some of which may be better captured by accelerometer-measured total PA rather than MVPA.

Third, the participants in our study had retired relatively recently but the sample was drawn from a 12-month activity-tracker based physical activity intervention [46]. This is likely to have introduced selection bias as the participants must have been willing to take part in a 12-month intervention. Based on non-respondent analyses, the participants were more likely to have a high occupational background when compared with non-participants [46]. Furthermore, the recruitment was focused on recent retirees from the public sector in Finland, which resulted in a sample with a high proportion of women. Due to the low number of men, we were not able to assess the potentially different associations with the meaning dimensions of physical activity and physical activity across genders. Furthermore, although the main analyses were adjusted with gender, the observed results are likely to be biased due to unbalanced gender distributions. In addition, the sample of the current study can be considered relatively healthy and physically active as evidenced by both self-reported and accelerometer-measured metrics of physical activity. The orientation towards recreational physical activity in general is likely to determine the extent to which physical activity provides a meaningful context for improving well-being, attaining new skills, meeting challenges, or social engagement. Thus, the generalizability of our findings is mainly limited to healthy recently retired women. Future studies are required to assess the robustness of our findings in a more representative sample of recent retirees. Nevertheless, our sample was diverse in terms of occupational backgrounds, which allowed us to compare the relative importance of different physical activity meaning dimensions across occupational background categories, albeit the generalizability is limited by the low number of men.

## 5. Conclusions

Our findings suggest that promotion of recreational physical activity among recent retirees should be mainly focused on maintaining health and fitness, on the positive immediate emotional outcomes of physical activity engagement, and on supporting social belonging through recreational physical activity, as these were found to be positively associated with changes in self-reported and accelerometer-measured physical activity over 12 months. In addition, the importance of meanings attributed to physical activity among recent retirees was observed to vary across occupational backgrounds. In particular, participants with a low occupational background attributed higher importance for meanings of physical activity associated with mental well-being. Further quantitative research on the facilitators of recreational physical activity with representative samples of recent retirees is needed, particularly with a focus on the potentially varying importance of the different preferences and facilitators of physical activity among recent retirees across socioeconomical backgrounds. Based on our findings, the meaning-based PAR approach appears as a promising tool for identifying the facilitators of physical activity among recent retirees.

## Figures and Tables

**Table 1:** Participant characteristics (*n* = 230).

	%
**Gender**	
Man	17
Woman	83
Occupational background	
High	38
Middle	27
Low	35
**Self-rated health**	
Good	46
Rather good	37
Average	17
	Mean (SD)
Age, years	65.2 (1.1)
Body Mass Index, kg/m^2^	27.2 (4.8)
**Self-reported weekly leisure-time physical activity, hours**	
Baseline	5.7 (2.9)
12-month follow-up	6.3 (3.0)
**Accelerometer-measured daily total physical activity, hours**	
Baseline	4.6 (1.5)
12-month follow-up	4.5 (1.6)
**Accelerometer-measured daily moderate-to-vigorous physical activity, minutes**	
Baseline	53.3 (29.6)
12-month follow-up	50.7 (28.9)

**Table 2:** The final solution of the exploratory factor analysis with eight meaning dimensions and 32 retained items with rotated factor loadings (loadings below 0.50 omitted) and extracted communalities (h^2^).

Item	F1	F2	F3	F4	F5	F6	F7	F8	h^2^
Attaining trendy image	0.813								0.730
Trendy physical activity location	0.754								0.655
Trendy equipment	0.745								0.576
Trendy sport	0.659								0.639
Technical gear/equipment	0.522								0.519
Alleviating stress		0.752							0.447
Developing self-control		0.749							0.624
Psychological growth		0.719							0.569
Increasing confidence		0.672							0.626
Own time		0.610							0.484
Unwinding		0.593							0.556
Mental balance		0.560							0.380
Improving fitness			0.842						0.673
Maintaining health			0.655						0.452
Physical exertion			0.595						0.624
Joy				0.640					0.509
Restoration				0.590					0.540
Feeling good				0.534					0.553
Relaxation				0.518					0.501
Being in a group					0.851				0.625
Sense of belonging					0.717				0.669
Like-minded others					0.644				0.560
Cooperation/encouragement					0.584				0.616
Learning new skills						0.606			0.599
New experiences						0.602			0.522
Versatility						0.570			0.482
Brisk action							0.722		0.552
Solitary exercise							0.503		0.495
Sense of competence							0.502		0.520
Aiming for better performances							0.501		0.452
Proximity of physical activity location								0.676	0.529
Affordability of physical activity								0.587	0.581
Initial eigenvalue	13.39	4.11	2.35	1.97	1.67	1.55	1.25	1.14	
Initial variance explained	29.76	9.13	5.21	4.39	3.72	3.44	2.78	2.50	
Cronbach alpha	0.86	0.85	0.75	0.79	0.84	0.76	0.72	0.65	

The identified meaning dimensions were titled as follows: F1: Trends and Status; F2: Mental Well-Being; F3: Physical Fitness; F4: Positive Mood; F5: Belonging; F6: New Experiences; F7: Achievement; F8: Practical Facilitators.

**Table 3:** Means and standard deviations of the importance of physical activity meaning dimensions (range 1–5) across gender and occupational background ranked by their overall importance with *p*-values and effect size (d, ω^2^).

Variable, Mean (SD)	Gender					Occupational Background				
	Overall *n* = 230	Women *n* = 191	Men *n* = 39	*p* ^1^	*d*	High *n* = 88	Middle *n* = 62	Low *n* = 80	*p* ^2^	ω^2^
Positive Mood	4.35 (0.58)	4.39 (0.58)	4.16 (0.52)	0.018	0.42	4.30 (0.61)	4.35 (0.59)	4.42 (0.53)	0.404	0.00
Physical Fitness	4.28 (0.59)	4.28 (0.59)	4.30 (0.55)	0.847	0.03	4.27 (0.58)	4.30 (0.66)	4.28 (0.54)	0.957	0.01
Practical Facilitators	3.70 (0.85)	3.80 (0.81)	3.22 (0.89)	<0.001	0.70	3.50 (0.81) ^a^	3.74 (0.96)	3.89 (0.77) ^a^	0.011	0.03
Mental Well-Being	3.41 (0.73)	3.47 (0.73)	3.13 (0.67)	0.007	0.48	3.18 (0.77) ^a^	3.37 (0.71) ^b^	3.70 (0.58) ^a,b^	<0.001	0.09
New Experiences	3.41 (0.78)	3.43 (0.79)	3.28 (0.73)	0.266	0.20	3.24 (0.82) ^a^	3.46 (0.83)	3.54 (0.67) ^a^	0.036	0.02
Belonging	3.22 (0.88)	3.30 (0.88)	2.86 (0.81)	0.005	0.50	3.04 (0.95) ^a^	3.22 (0.87)	3.42 (0.78) ^a^	0.023	0.02
Achievement	2.80 (0.78)	2.83 (0.78)	2.63 (0.79)	0.150	0.25	2.53 (0.78) ^a^	2.75 (0.61) ^b^	3.13 (0.79) ^a,b^	<0.001	0.10
Trends and Status	1.74 (0.76)	1.75 (0.78)	1.69 (0.70)	0.656	0.08	1.53 (0.70) ^c^	1.50 (0.47) ^d^	2.16 (0.85) ^c,d^	<0.001 ^3^	

^1^ Independent samples *t*-test; ^2^ ANOVA; ^3^ Kruskal–Wallis test. ^a–d^ Means with the same superscript are significantly different at *p* < 0.05 level after Bonferroni correction ^a,b^, or Dwass, Steel, Critchlow–Fligner method for multiple comparisons ^c,d^.

**Table 4:** The associations between physical activity meaning dimensions and self-reported leisure-time physical activity (LTPA) over 12 months (Pearson correlation coefficients for correlations at baseline (*r*) and covariate adjusted general linear models (B, 95% CI, F, *p*) with change in R^2^ with reference to the base model with covariates (ΔR^2^)).

		Self-Reported LTPA at Baseline ^1^				Changes in Self-Reported LTPA over 12 Months ^2^			
Base Model with covariates		F_7,222_ = 3.61, *p* = 0.0011, R^2^ = 0.102				F_9,213_ = 6.90, *p* < 0.0001, R^2^ = 0.226			
	*r*	B (95% CI)	F	*p*	ΔR^2^	B (95% CI)	F	*p*	ΔR^2^
Positive Mood	0.13 *	0.13 (0.00 to 0.26)	3.98	0.047	0.016	0.18 (0.06 to 0.30)	8.69	0.004	0.030
Physical Fitness	0.17 **	0.18 (0.05 to 0.31)	7.95	0.005	0.031	0.18 (0.06 to 0.30)	8.40	0.004	0.030
Practical Facilitators	0.07	0.06 (−0.08 to 0.19)	0.72	0.398	0.003	0.09 (−0.04 to 0.21)	1.90	0.170	0.007
Mental Well-Being	0.11 ^a^	0.14 (0.01 to 0.28)	4.59	0.033	0.018	0.00 (−0.12 to 0.14)	0.02	0.884	0.000
New Experiences	0.19 **	0.21 (0.08 to 0.33)	10.27	0.002	0.040	0.05 (−0.08 to 0.18)	0.60	0.439	0.002
Belonging	0.14 *	0.17 (0.03 to 0.30)	6.04	0.015	0.024	0.14 (0.01 to 0.27)	4.75	0.030	0.017
Achievement	0.10	0.11 (−0.02 to 0.25)	2.73	0.099	0.011	0.06 (−0.07 to 0.19)	0.73	0.395	0.003
Trends and Status	0.10	0.08 (−0.05 to 0.22)	1.43	0.233	0.006	0.05 (−0.08 to 0.19)	0.61	0.435	0.002

** *p* < 0.01, * *p* < 0.05, ^a^ *p* < 0.10. ^1^ adjusted with age, body mass index, gender, occupational background, self-rated health. ^2^ adjusted with age, body mass index, gender, occupational background, self-rated health, intervention group, baseline level.

**Table 5:** The associations between physical activity meaning dimensions and accelerometer-measured total physical activity (Total PA) over 12 months (Pearson correlation coefficients for correlations at baseline (*r*) and covariate adjusted general linear models (B, 95% CI, F, *p*) with change in R^2^ with reference to the base model with covariates (ΔR^2^)).

		Total PA at Baseline ^1^				Changes in Total PA over 12 Months ^2^			
Base Model with covariates		F_8,221_ = 3.03, *p* = 0.003, R^2^ = 0.099				F_10,213_ = 28.22, *p* < 0.0001, R^2^ = 0.570			
	*r*	B (95% CI)	F	*p*	ΔR^2^	B (95% CI)	F	*p*	ΔR^2^
Positive Mood	0.06	0.00 (−0.13 to 0.13)	0.00	0.968	0.000	0.09 (0.00 to 0.18)	3.51	0.062	0.007
Physical Fitness	0.04	0.01 (−0.12 to 0.14)	0.04	0.844	0.000	0.11 (0.02 to 0.20)	5.40	0.021	0.011
Practical Facilitators	0.08	−0.02 (−0.15 to 0.12)	0.06	0.812	0.000	0.15 (0.06 to 0.24)	10.10	0.002	0.020
Mental Well-Being	0.15 *	0.09 (−0.05 to 0.23)	1.74	0.189	0.007	0.05 (−0.04 to 0.15)	1.26	0.263	0.003
New Experiences	0.11 ^a^	0.06 (−0.07 to 0.19)	1.41	0.236	0.004	0.05 (−0.04 to 0.14)	1.07	0.302	0.002
Belonging	0.05	−0.01 (−0.15 to 0.13)	0.02	0.886	0.000	0.12 (0.03 to 0.22)	6.36	0.012	0.013
Achievement	0.13 *	0.08 (−0.05 to 0.22)	1.46	0.228	0.006	0.00 (−0.10 to 0.09)	0.00	0.980	0.000
Trends and Status	0.05	0.01 (−0.13 to 0.16)	0.04	0.838	0.000	−0.01 (−0.11 to 0.09)	0.05	0.815	0.000

* *p* < 0.05, ^a^ *p* < 0.10. ^1^ adjusted with age, body mass index, gender, occupational background, self-rated health, wear time. ^2^ adjusted with age, body mass index, gender, occupational background, self-rated health, intervention group, baseline level, difference in wear time t12–t0.

## Data Availability

The data presented in this study are available on reasonable request from the corresponding author. The data are not publicly available due to confidential personal details and health information.

## Supplementary Materials

The following supporting information can be downloaded at: https://www.mdpi.com/article/10.3390/ijerph192315567/s1, Table S1: Individual items included in the 54-item inventory assessing the importance of meanings attributed to physical activity; Table S2: The associations between physical activity meaning dimensions and accelerometer-measured moderate-to-vigorous physical activity (MVPA) over 12 months (Pearson correlation coefficients for zero-order correlations at baseline (*r*) and covariate adjusted general linear models (B, 95% CI, F, *p*) with change in R^2^ with reference to the base model with covariates (ΔR^2^)).
