# Supplementary material for: Meanings Attributed to Physical Activity and Changes in Self-Reported and Accelerometer-Measured Physical Activity among Recent Retirees
Source: Int J Environ Res Public Health. 2022 Nov 23;19(23):15567. doi: 10.3390/ijerph192315567 (PMC9736060; doi:10.3390/ijerph192315567)
Supplement: Supplementary file 1 [file ijerph-19-15567-s001.zip › ijerph-1956017-supplementary.pdf]

Supplementary Table S1. Individual items included in the 54-item inventory assessing the importance of meanings attributed to physical activity.

Item	n	Mean	Standard deviation	Rated as fairly or very important (%)
Maintaining health	230	4.70	0.57	96
Nature	230	4.56	0.73	95
Restoration	229	4.44	0.66	96
Feeling good	230	4.40	0.78	92
Joy	221	4.39	0.77	91
Improving fitness	228	4.29	0.74	90
Finding balance	230	4.25	0.81	84
Relaxation	229	4.19	0.75	86
Making social contacts	229	4.14	0.70	84
Regularity	230	3.93	0.87	75
Knowing one's own body	229	3.89	0.81	75
Physical exertion	229	3.86	0.83	77
Spending time with significant others	230	3.79	1.05	70
Proximity of physical activity location	229	3.76	0.95	70
Regular program	230	3.73	0.98	69
Familiar sport	228	3.72	0.87	64
Attaining muscle	228	3.71	0.94	64
Fun/play	227	3.71	0.96	65
Joy of success	229	3.69	0.97	66
Own time	229	3.68	0.95	62
Weight maintenance	229	3.66	1.11	64
Unwinding	230	3.65	0.94	64
Affordability of physical activity	228	3.63	1.02	61
Versatility	229	3.56	0.92	59
Monitoring development	229	3.52	0.92	58
New experiences	228	3.48	0.94	54
Like-minded others	227	3.44	1.09	58
Mental stimulation	230	3.35	0.94	45
Sense of belonging	229	3.34	1.01	45
Psychological growth	229	3.33	1.01	43
Alleviating stress	229	3.29	1.10	50
Learning new skills	226	3.18	1.00	39
Instruction from others	229	3.13	1.09	41
Being in a group	229	3.06	1.14	37
Sense of competence	230	3.05	1.07	35
Cooperation/encouragement	229	3.05	1.07	38
Facilities of a sport location	230	3.02	1.06	36
Increasing confidence	229	2.94	1.04	27
Aiming for better performances	229	2.90	1.04	29
Improving appearance	230	2.78	1.12	30
Developing self-control	228	2.74	1.14	24
Brisk action	229	2.72	1.10	24
Doing physical activity alone	230	2.52	1.01	13
Adopting a different role	229	2.28	1.05	11
Minimizing failure	230	2.26	1.06	10
Pushing the limits	227	2.22	1.09	13
Technical gear/equipment	229	2.13	1.12	13
Risk/excitement	228	1.90	1.00	6
Trendy equipment	228	1.68	0.92	4
Trendy sport	229	1.68	0.84	2
Trendy physical activity location	227	1.63	0.98	5
Competition	229	1.58	0.86	2

Attaining trendy image	229	1.57	0.84	2
Success/Winning	228	1.50	0.79	1

Supplementary Table S2. The associations between physical activity meaning dimensions and accelerometer-measured moderate-to-vigorous physical activity (MVPA) over 12 months (Pearson correlation coefficients for zero-order correlations at baseline (*r*) and covariate adjusted general linear models (B, 95% CI, F, p) with change in R^2 with reference to the base model with covariates (ΔR^2)).

	**MVPA at baseline**[1]					**Changes in total MVA over 12 months**[2]			
**Base Model with covariates**	$F_{8,221}$ = 2.90, p=0.0042, R2=0.095					$F_{10,213}$=23.68, p<.0001, R^2=0.526			
	r	B (95% CI)	F	*p*	ΔR^2	B (95% CI)	F	*p*	ΔR^2
**Positive Mood**	-0.01	-0.02 (-0.15 to 0.11)	0.10	0.757	0.000	0.06 (-0.04 to 0.15)	1.47	0.226	0.003
**Physical Fitness**	0.07	0.06 (-0.07 to 0.19)	0.91	0.304	0.004	0.09 (0.00 to 0.19)	3.55	0.061	0.007
**Practical Facilitators**	0.01	-0.02 (-0.16 to 0.11)	0.12	0.731	0.000	0.04 (-0.06 to 0.14)	0.66	0.416	0.001
**Mental Well-Being**	0.10	0.11 (-0.02 to 0.25)	2.61	0.107	0.010	0.02 (-0.08 to 0.12)	0.15	0.703	0.000
**New Experiences**	-0.02	0.00 (-0.14 to 0.12)	0.01	0.935	0.000	0.02 (-0.07 to 0.12)	0.23	0.633	0.000
**Belonging**	-0.06	-0.02 (-0.15 to 0.12)	0.08	0.801	0.000	0.03 (-0.07 to 0.13)	0.37	0.544	0.000
**Achievement**	0.07	0.09 (-0.05 to 0.22)	1.54	0.216	0.006	0.01 (-0.09 to 0.11)	0.05	0.822	0.000
**Trends & Status**	-0.03	-0.02 (-0.16 to 0.12)	0.11	0.745	0.000	0.00 (-0.10 to 0.10)	0.00	0.995	0.000

[1] adjusted with age, body mass index, gender, occupational background, self-rated health, wear time. [2] adjusted with age, body mass index, gender, occupational background, self-rated health, intervention group, baseline level, difference in wear time t12 – t0.
